# Clinical Prediction Model for Screening Acute Ischemic Stroke Patients With More Than 10 Cerebral Microbleeds

**DOI:** 10.3389/fneur.2022.833952

**Published:** 2022-04-07

**Authors:** Yifan Li, Haifeng Gao, Dongsen Zhang, Xuan Gao, Lin Lu, Chunqin Liu, Qian Li, Chunzhi Miao, Hongying Ma, Yongqiu Li

**Affiliations:** ^1^Department of Neurology, Xuanwu Hospital of Capital Medical University, Beijing, China; ^2^Department of Neurology, Tangshan Gongren Hospital, Tangshan, China

**Keywords:** cerebral microbleeds, prediction model, cerebral small vessel disease, intravenous thrombolysis, hemorrhagic transformation

## Abstract

**Background:**

Hemorrhagic transformation is one of the most serious complications in intravenous thrombolysis. Studies show that the existence of more than 10 cerebral microbleeds is strongly associated with hemorrhagic transformation. The current study attempts to develop and validate a clinical prediction model of more than 10 cerebral microbleeds.

**Methods:**

We reviewed the computed tomography markers of cerebral small vessel diseases and the basic clinical information of acute ischemic stroke patients who were investigated using susceptibility weighted imaging from 2018 to 2021. A clinical prediction model of more than 10 cerebral microbleeds was established. Discrimination, calibration, and the net benefit of the model were assessed. Finally, a validation was conducted to evaluate the accuracy and stability of the model.

**Results:**

The multivariate logistic regression model showed hypertension, and some computed tomography markers (leukoaraiosis, lacunar infarctions, brain atrophy) were independent risk factors of more than 10 cerebral microbleeds. These risk factors were used for establishing the clinical prediction model. The area under the receiver operating characteristic curve (AUC) was 0.894 (95% CI: 0.870–0.919); Hosmer–Lemeshow chi-squared test yielded χ^2^ = 3.946 (*P* = 0.862). The clinical decision cure of the model was higher than the two extreme lines. The simplified score of the model ranged from 0 to 12. The model in the internal and external validation cohort also had good discrimination (AUC 0.902, 95% CI: 0.868–0.937; AUC 0.914, 95% CI: 0.882–0.945) and calibration (*P* = 0.157, 0.247), and patients gained a net benefit from the model.

**Conclusions:**

We developed and validated a simple scoring tool for acute ischemic stroke patients with more than 10 cerebral microbleeds; this tool may be beneficial for paradigm decision regarding intravenous recombinant tissue plasminogen activator therapy of acute ischemic stroke.

## Introduction

Cerebral microbleeds (CMBs) are small, round, or oval hypointense lesions found on susceptibility-weighted imaging (SWI) as subclinical hemosiderin deposits due to hemorrhage from microvascular lesions (1). The prevalence rate of cerebral microbleeds ranges from 15 to 71% (2, 3) in patients with acute ischemic stroke (AIS) and 50-80% in patients with hemorrhagic stroke (4). Symptomatic intracranial hemorrhage (sICH) caused by thrombolytic therapy is associated with CMB, and a heavier burden of CMB imparts a higher risk of hemorrhagic transformation (HT) (5–7). A CMB burden of more than 10 on baseline neuroimaging before intravenous thrombolytic therapy was independently associated with symptomatic hemorrhagic transformation, which ranges from 28.6 to 46.9% (8–11). Schlemm et al. also found that intravenous thrombolysis was associated with higher mortality in patients with >10 CMBs (8).

sICH is a severe therapeutic complication that greatly impedes functional recovery and increases mortality (12). sICH is observed in approximately 5% of patients treated with intravenous thrombolysis (13). Thus, early prediction of sICH before thrombolytic therapy is extremely necessary for guiding precise treatment paradigm decisions. In the event of acute cerebral infarction, thrombolytic therapy should be performed as soon as possible, except in patients with contraindications. SWI (14) is the preferred deterministic diagnostic technique for CMB, but it is not possible to conduct SWI before thrombolysis (15), as this would lead to a delay in the short time frame during which treatments should be initiated, thus violating the “time is brain” principle; furthermore, this cannot be performed in primary hospitals. Computed tomography (CT) scans must be completed before thrombolytic therapy for patients with AIS and can also reveal imaging manifestations of some cerebral small vessel disease (CSVD) such as leukoaraiosis, brain atrophy, lacunar infarctions, and recent small infarctions (16). As a CSVD marker, CMBs are related to the CSVD burden, which may be indicated by the number of CMBs. However, the relationship between the number of CMBs and CT markers of CSVD is unknown.

The purpose of this study was to develop and validate a practical and easily implemented operating clinical prediction model (CPM) to predict the probability of the presence of >10 CMBs on the basis of easily collected information such as CT markers of CSVD and past medical history.

## Materials and Methods

### Participants

This study was conducted at the Tangshan Gongren Hospital and Tangshan Nanhu' Hospital. Patients with AIS who underwent SWI and head CT scans during hospitalization from January 2018 to December 2021 were recruited. The inclusion criteria were AIS without intravenous thrombolytic therapy and patient age of ≥18 years. The exclusion criteria were as follows: (1) coagulation disorders; (2) arteriovenous malformation; (3) moyamoya disease; (4) previous intracerebral hemorrhage and subarachnoid hemorrhage; (5) cerebral trauma; (6) infarct size greater than 2/3 of the territory of the middle cerebral artery supply; (7) previous history of anticoagulant therapy; (8) heart, liver, or kidney failure; or (9) active internal bleeding.

Demographic information (including sex and age), self-reported history of disease (such as hypertension, stroke, and diabetes), and unhealthy lifestyle factors were noted retrospectively.

This study was approved by the Ethics Committee of Tangshan Gongren Hospital (Approval Number: GRYY-LL-KJ2021-K93).

### Imaging Examination

All patients underwent a brain magnetic resonance imaging (MRI) scan (Philips Achieva 1.5T) with a 12-channel head coil and a brain CT scanning of the brain with a 64-detector row (Siemens Germany). Scanning sequences included both MRI and SWI sequences.

### Imaging Assessment

CMBs (17) present as small, rounded or circular, well-circumscribed, hypointense parenchymal lesions as large as 2–10 mm in size on the SWI. Participants were divided into two groups according to the number of CMBs: 0-10 and >10.

Imaging manifestations of CSVD on CT scanning were determined as follows. Leukoaraiosis was evaluated according to the Blennow scale (18) (scores ranged from 0 to 3). The global scale of cortical atrophy (19) was used to assess the degree of brain atrophy based on a five-point scale (0 = “none,” 1 = “mild,” 2 = “moderate,” and 3 = “severe”). We defined lacunar infarctions as round or ovoid hypodense lesions of 3-20 mm diameter in the basal ganglia, deep white matter, cerebellum, or pons on the CT scan. Lacunar infarctions were scored as follows: 0, no lacunar lesion; 1, 1–5 lacunar infarctions; 2, 5–10 lacunar infarctions; and 3, >10 lacunar infarctions (12).

The images were interpreted cooperatively by three neurologists who were blinded regarding the relationship of CT scan characteristics, CMBs on SWI, and clinical information. The three neurologists consisted of one neuroimagist and two neurologist clinicians.

### Statistical Analysis

In the development and validation cohorts, we compared data between patients with 0–10 and >10 CMBs. Continuous variables are presented as means and standard deviations. Between-group comparisons were performed using Student's *t*-test if data were normally distributed and the Mann–Whitney test if data were not normally distributed. Categorical variables are presented as numbers and frequencies. We compared categorical variables between groups with the χ^2^ test or Fisher's exact test.

In the development cohort, univariate logistic regression was used to examine the relationship between a single covariate, such as CT scan characteristics and other clinical information, and the existence of >10 CMBs as indicated by SWI. Multivariate logistic regression was used to investigate independent risk factors using all risk factors selected. Independent risk factors were applied to construct the CPM for >10 CMBs, based on coefficients and odds ratios with 95% confidence intervals (CIs).

The clinical predictor efficiency of the CPM was evaluated by the following steps. First, we assessed discrimination using Harrell's C statistic, which was equivalent to the area under the receiver operator characteristic (ROC) curve (AUC). An AUC of 0.5 indicates no discrimination, whereas an AUC of 1.0 indicates perfect discrimination. Second, calibration was carried out to evaluate the accuracy of the model. The goodness-of-fit based on the Hosmer–Lemeshow chi-squared test of the CPM was performed for assessing the fit of the model. A *P*-value ≥ 0.05 was determined to show goodness-of-fit. Third, decision curve analysis (DCA) was generated on the basis of the multivariate prediction model using R software (version 4.0.3) and was used to evaluate the net benefit of the model.

*P*-values were two-sided, and values of <0.05 were considered statistically significant. All data were analyzed using SPSS software (version 22.0, IBM company, New York, USA).

## Results

### Characteristics of the Development and Validation Cohorts

**Figure 1:**
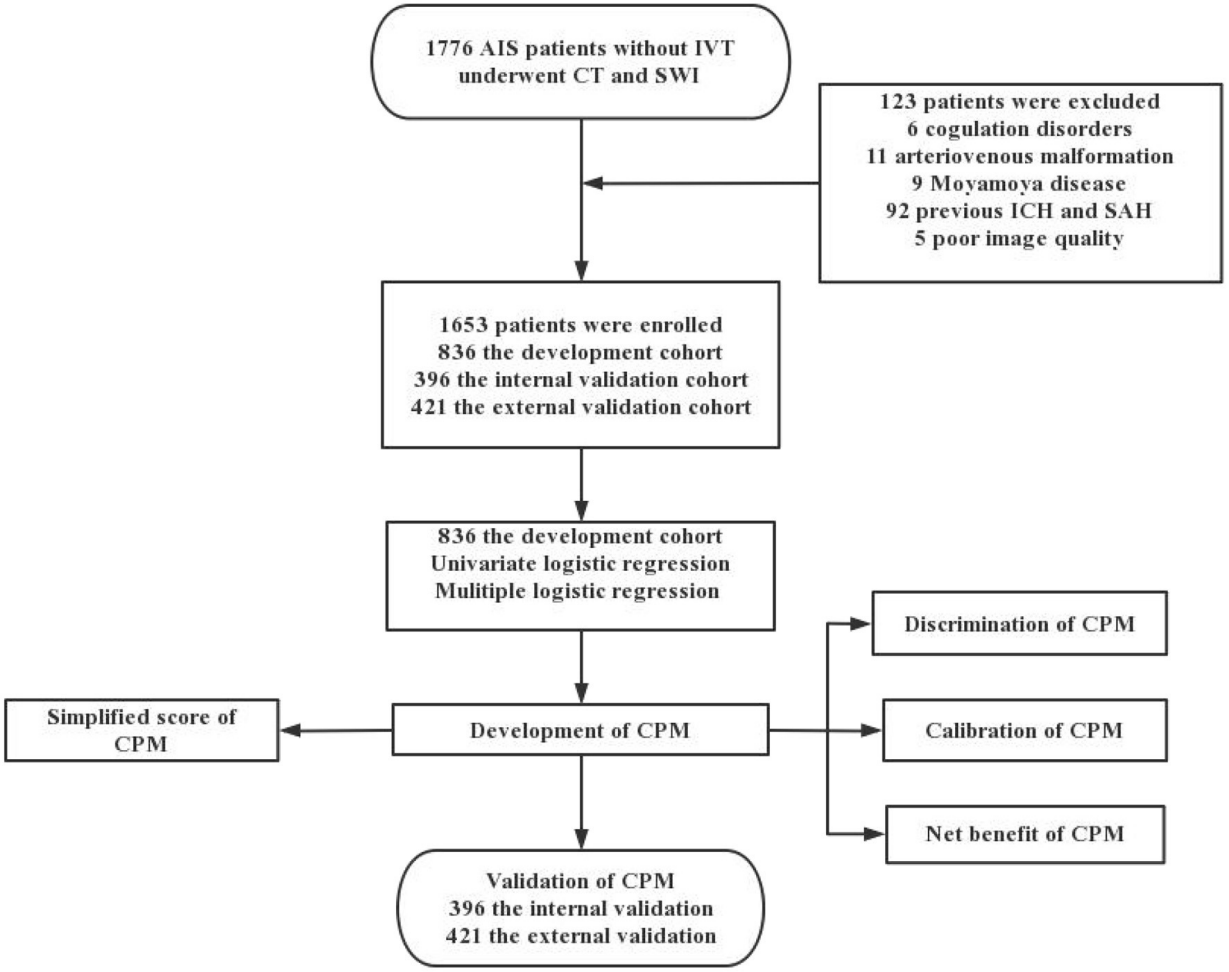
Flow chart of the study.

In total, 1,776 patients with AIS underwent head CT and SWI, 123 of whom were excluded, leaving 1,653 patients enrolled in the study. Among them, 836 patients from Tangshan Workers' Hospital were selected as the development cohort from January 2018 to December 2019 and 396 patients were selected as the internal validation cohort from January 2020 to December 2020. Four hundred and twenty one patients from Nanhu' Hospital were enrolled for the external validation cohort from October 2020 to December 2021 (Figure 1). Of the included patients, 483 exhibited >10 CMBs, accounting for 31.58, 25.00, and 28.50% of patients in the development, internal and external cohorts, respectively. Characteristics of the development and validation cohorts are shown in Table 1. There were statistically significant differences between the 0–10 CMB and >10 CMB groups in terms of age, history of hypertension, leukoaraiosis, brain atrophy, and lacunar infarction. The proportion of patients with stroke history in the >10 CMB group was higher than that in the 0–10 CMB group (*P* = 0.035 in the internal validation cohort), but there were no differences between the development and external validation cohorts.

**Table 1:** Characteristics in development cohort and validation cohort.

**Variable**	**Development cohort [n (%)]**			**Internal validation cohort [n (%)]**			**External validation cohort [n (%)]**		
	**0-10CMBs**	**>10 CMBs**	***P*-value**	**0-10CMBs**	**>10 CMBs**	***P*-value**	**0-10CMBs**	**>10 CMBs**	***P*-value**
	**[572 (68.42)]**	**[264 (31.58)]**		**[297 (75.00)]**	**[99 (25.00)]**		**[301 (71.50)]**	**[120 (28.50)]**	
Age (X ± s)	59.99 ± 10.68	63.89 ± 10.48	<0.001	60.61 ± 10.97	64.10 ± 9.81	0.005	61.23 ± 12.82	62.26 ± 10.85	0.445
Male [*n* (%)]	384 (67.13)	184 (69.70)	0.369	186 (62.63)	69 (69.70)	0.204	181 (60.13)	84 (70.00)	0.058
**Medical history**
Diabetes [*n* (%)]	101 (17.65)	56 (21.21)	0.479	80 (26.94)	20 (20.20)	0.183	84 (27.91)	25 (20.83)	0.127
Hypertension [*n* (%)]	378 (66.08)	214 (81.44)	<0.001	184 (61.95)	88 (88.89)	<0.001	181 (60.13)	103 (85.83)	<0.001
Drinking [*n* (%)]	138 (24.13)	81 (30.68)	0.061	73 (24.58)	28 (28.28)	0.558	47 (15.61)	26 (21.67)	0.140
Coronary heart disease [*n* (%)]	129 (22.55)	30 (11.36)	0.954	50 (16.84)	16 (16.16)	0.876	45 (14.95)	26 (21.67)	0.104
Stroke [*n* (%)]	155 (27.10)	95 (35.98)	0.083	68 (22.90)	35 (35.35)	0.035	78 (25.91)	42 (35.00)	0.072
**Characteristics of CT**
Leukoaraiosis			<0.001			<0.001			<0.001
0–1 score [*n* (%)]	503 (89.94)	101 (38.26)		255 (85.86)	26 (26.26)		233 (77.41)	18 (15.00)	
2 score [*n* (%)]	55 (9.62)	89 (33.71)		39 (13.13)	50 (50.50)		62 (20.60)	60 (50.00)	
3 score [*n* (%)]	14 (2.45)	74 (28.03)		3 (1.01)	23 (23.23)		6 (1.99)	42 (35.00)	
Brain atrophy			<0.001			<0.001			<0.001
0–1 score [*n* (%)]	477 (83.39)	60 (22.73)		228 (76.77)	19 (19.19)		194 (64.45)	22 (18.33)	
2 score [*n* (%)]	53 (9.27)	56 (21.21)		46 (15.49)	27 (27.27)		64 (21.26)	29 (24.17)	
3 score [*n* (%)]	42 (7.34)	148 (56.06)		23 (7.74)	53 (53.54)		43 (14.29)	69 (57.50)	
Lacunar infarction			<0.001			<0.001			<0.001
0–1 score [*n* (%)]	401 (70.10)	66 (25,00)		228 (76.77)	20 (20.20)		231 (76.74)	32 (26.67)	
2 score [*n* (%)]	122 (21.33)	101 (38.26)		52 (17.51)	23 (23.23)		51 (16.94)	23 (19.17)	
3 score [*n* (%)]	49 (8.57)	97 (36.74)		17 (7.74)	56 (56.57)		19 (6.31)	65 (54.17)	

### CPM Development

Univariate risk factors for the presence of >10 CMBs are summarized in Table 2; having >10 CMBs was associated with age, hypertension, leukoaraiosis, brain atrophy, and lacunar infarctions and was closely related to the severity of leukoaraiosis, brain atrophy, and lacunar infarction. In contrast, sex, diabetes, alcohol consumption, coronary heart disease, and stroke were independent of >10 CMBs. Multivariate analyses were performed using the risk factors determined in the univariate analysis, such as age, hypertension, leukoaraiosis, brain atrophy, and lacunar infarction. Hypertension, leukoaraiosis, brain atrophy, and lacunar infarction were revealed as significant independent factors for >10 CMBs, whereas age was not an independent factor. The multivariate logistic regression model was established as follows (see Table 3):

**Table 2:** Univariate analysis of risk factors for more than 10 CMBs.

**Variable**	**Subgroup**	**B**	**S.E**	**Wald/χ^2^**	**Univariate analysis**		
					**OR**	**95%CI**	***P*-value**
Age		0.036	0.007	22.977	1.036	1.021–1.051	<0.001
Male		0.145	0.161	0.805	1.156	0.843–1.585	0.369
Diabetes		0.134	0.190	0.502	1.144	0.789–1.659	0.479
Hypertension		0.787	0.180	19.069	2.197	1.543–3.127	<0.001
Drinking		0.311	0.166	3.509	1.338	0.986–1.888	0.061
Coronary heart disease		−0.047	0.186	0.064	0.954	0.662–1.374	0.800
Stroke		0.279	0.161	3.004	1.322	0.964–1.814	0.083
Leukoaraiosis				183.823			<0.001
	0–1 score				1.000		
	2 score	2.108	0.203	107.634	8.231	5.527–12.257	<0.001
	3 score	3.280	0.311	111.020	26.587	14.443-48.943	<0.001
Brain atrophy				239.549			<0.001
	0–1 score				1.000		
	2 score	2.797	0.233	88.913	9.000	5.700–14.210	<0.001
	3 score	3.305	0.223	220.633	27.257	17.622–42.159	<0.001
Lacunar infarction				144.484			<0.001
	0–1 score				1.000		
	2 score	1.625	0.189	74.103	5.080	3.509–7.354	<0.001
	3 score	2.477	0.220	126.567	11.904	7.732–18.326	<0.001

**Table 3:** Multiariable logistic regression analysis.

**Variable**	**Coefficients**	**S.E**	**Wald**	***P*-value**	**OR**	**95%CI**
Age	−0.011	0.010	1.107	0.293	0.989	0.970	1.009
Hypertension	0.589	0.246	5.723	0.017	1.802	1.112	2.919
Leukoaraiosis							
0-1 score			44.450	0.000			
2 score	1.090	0.264	17.074	0.000	2.975	1.774	4.990
3 score	2.251	0.364	38.244	0.000	9.489	4.652	19.376
Brain atrophy							
0-1 score			118.612	0.000			
2 score	1.303	0.275	22.446	0.000	3.681	2.147	6.311
3 score	2.696	0.248	117.812	0.000	14.823	9.109	24.120
Lacunar infarction							
0-1 score			23.425	0.000			
2 score	0.866	0.243	12.743	0.000	2.377	1.478	3.824
3 score	1.311	0.291	20.306	0.000	3.712	2.098	6.566
Constant	−2.554	0.652	15.368	0.000	0.079	-	-

Logit *P* = −2.554 + 0.589 × (hypertension) + 1.090 × (2 score, leukoaraiosis) + 2.251 × (3 score, leukoaraiosis) + 1.303 × (2 score, brain atrophy) + 2.696 × (3 score, brain atrophy) + 0.866 × (2 score, lacunar infarction) + 1.311 × (3 score, lacunar infarction).

Risk factor scoring was as follows: hypertension, yes = 1 and no = 0; leukoaraiosis, 0–1 = 0, 2 = 1, and 3 = 2; brain atrophy, 0–1 = 0, 2 = 1, and 3 = 2; and lacunar infarction, 0–1 = 0, 2 = 1, and 3 = 2.

### Simplified CPM Score

A simple scoring method was developed by assigning the independent risk factors a value expressed as a whole number. The value was obtained using coefficients as indicated in Table 3. The coefficients of leukoaraiosis, brain atrophy, and lacunar infarction were divided by the smallest coefficient of hypertension (0.589), the quotients and the divisor were converted into whole numbers according to the principle of rounding-up, and they were assigned values of corresponding independent risk factors. Assignment of independent risk factors in the model was as follows: hypertension was assigned as 1, a leukoaraiosis scale of 2 as 2, a leukoaraiosis scale of 3 as 4, moderate brain atrophy as 2, severe brain atrophy as 5, 2 lacunar infarctions as 1, and 3 lacunar infarctions as 2. The total scores ranged from 0 to 12, as summarized in Table 4.

**Table 4:** Simplified CPM.

**Variable in CPM**	**Score**
**Hypertension**
NO	0
YES	1
**Leukoaraiosis**
0-1 score	0
2 score	2
3 score	4
**Brain atrophy**
0-1 score	0
2 score	2
3 score	5
**Lacunar infarction** **0-1score** **0**
0-1 score	0
2 score	1
3 score	2

### CPM Assessment

**Figure 2:**
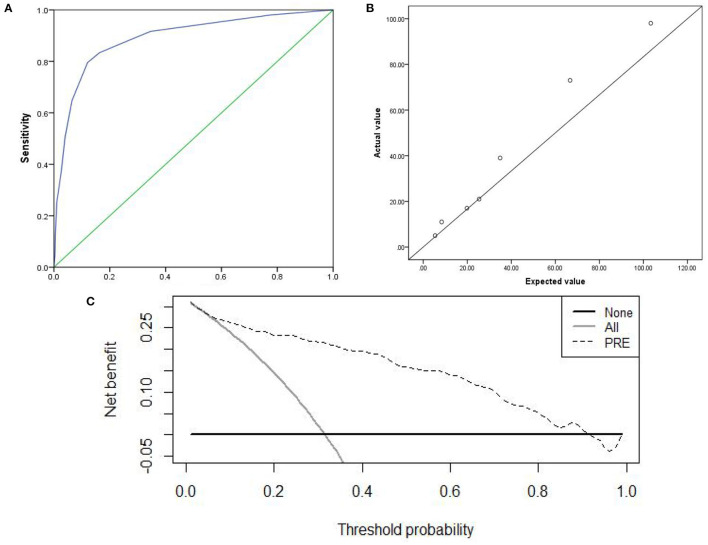
Discrimination, Calibration, clinical practicability of CPM were exhibited. **(A)** AUC of ROC, which indicated discrimination ability of the CPM; **(B)** Calibration scatter plots, which assessed calibration of the CPM; **(C)** DCA, which evaluated clinical practicability of the CPM.

Based on the multi-factor analysis, the prediction accuracy of the CPM was 84.3%; as this was more than 80%, this implied that the CPM score could be used for clinical practice. The AUC of the CPM was 0.894 (95%CI, 0.870–0.919); as this was larger than 0.5, this indicated the CPM has good discrimination (Figure 2A).

The χ^2^ value was 3.946 (*P* = 0.862) in the Hosmer–Lemeshow chi-squared test to assess the fit of the model. Calibration scatter plots are presented in Figure 2B. According to the scatter plot, values did not significantly deviate from the reference line, suggesting good discrimination and accuracy.

We used DCA to evaluate the clinical practicability of the CPM. The DCA of the CPM was higher than the two extreme lines, indicating that the CPM manifests practical clinical value (Figure 2C).

### CPM Internal and External Validation

**Figure 3:**
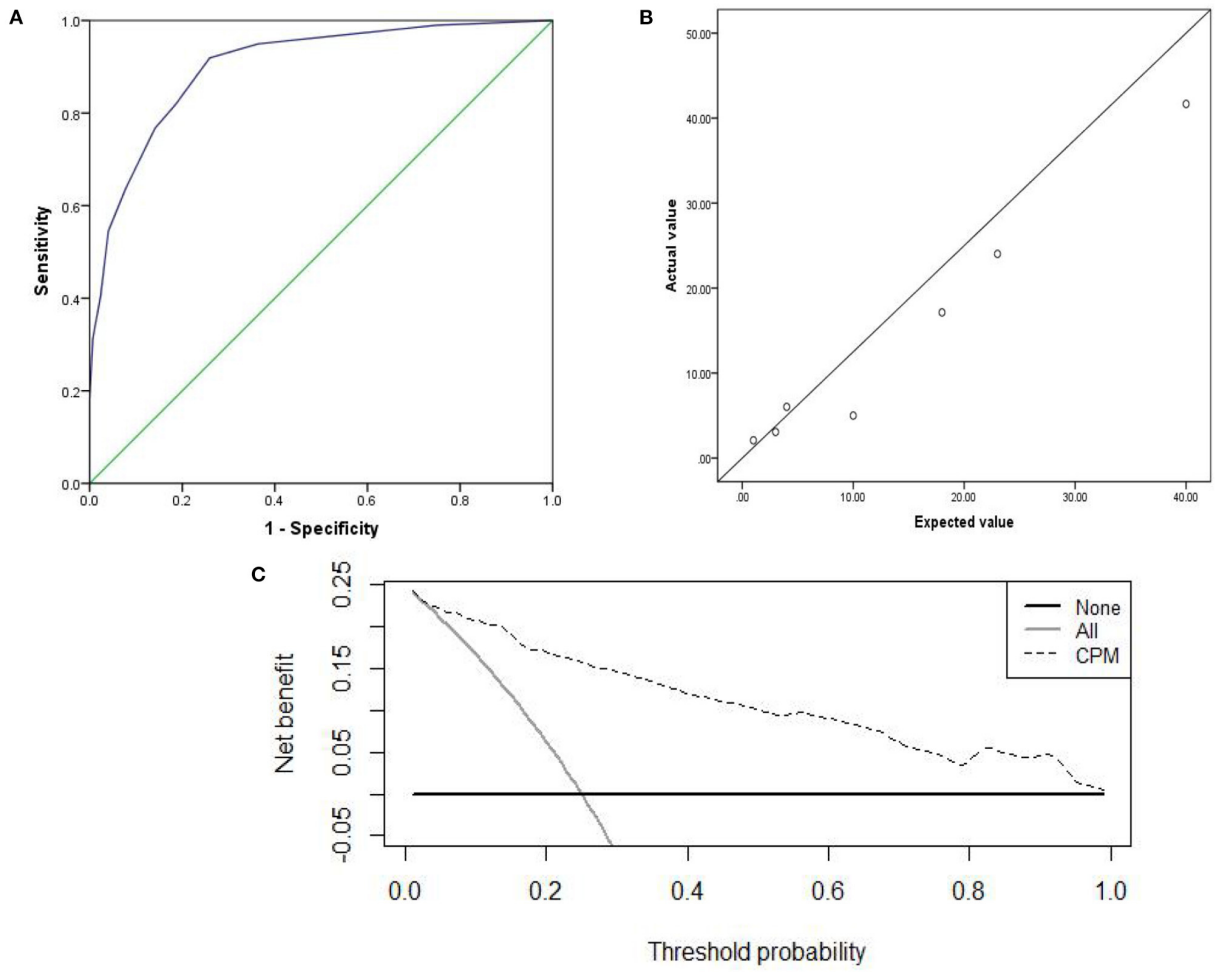
The internal validation: **(A)** AUC of the internal validation; **(B)** calibration scatter plots of the internal validation. **(C)** DCA of the internal validation.

**Figure 4:**
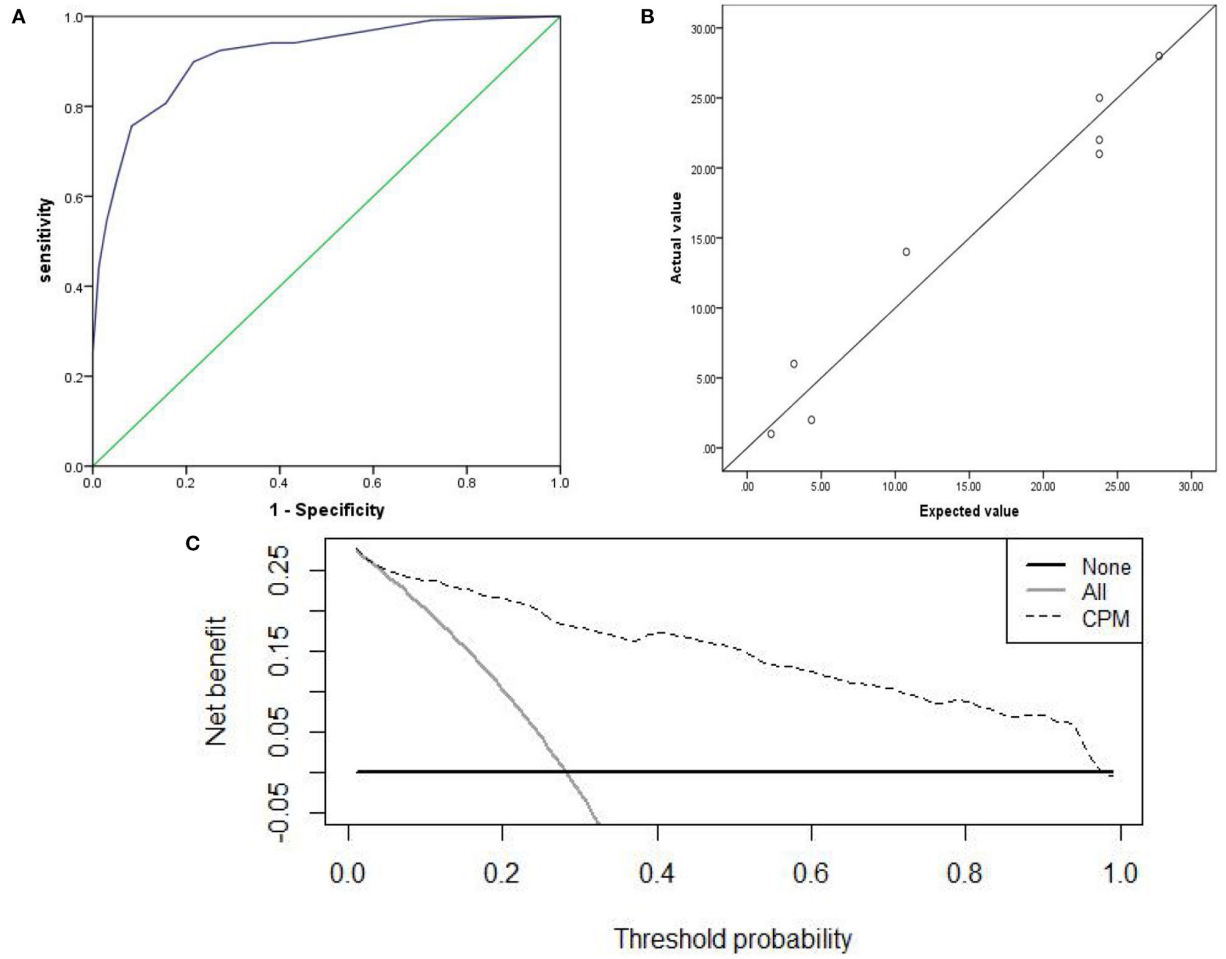
The external validation: **(A)** AUC of the external validation; **(B)** calibration scatter plots of the external validation. **(C)** DCA of the external validation.

The prediction accuracy of the internal and external validation were 85.1 and 87.1%. The AUC of the internal and external validation, which indicates discrimination ability, were 0.902 (95% CI, 0.868–0.937) and 0.914(95% CI, 0.882–0.945); the ROC curves were shown in Figures 3A, 4A. The CPM still achieved good discrimination in the internal and external validation.

The χ^2^ value in the Hosmer–Lemeshow test of the internal and external validation group was 7.992 (*P* = 0.157), and 7.878 (*P* = 0.247), as displayed in Figures 3B, 4B. The *P*-values were >0.05, indicating that the predicted observation values showed good consistency with the actual observation values. Accordingly, the use of the CPM accurately predicted individual outcomes when applied to the internal and external validation.

The DCA of the internal and external validation was also higher than the two extreme lines, indicating that the CPM has practical clinical value and can be beneficial in patients, as shown in Figures 3C, 4C.

### CPM Prediction Capability

All 836 subjects from the development cohort were enrolled into the predictive model scoring system for clinical analysis. According to the ROC curve, the cut-off point for the discrimination of >10 CMBs was 5, with a sensitivity and a specificity of 72.73 and 90.73%, respectively. The accuracy, positive predictive value, and negative predictive value of the CPM were 85.41, 78.36, and 87.82%, respectively. The sensitivity and specificity of CPM were 63.64 and 92.26%, respectively in the internal validation, 80.67 and 84.49% in the external validation (see Table 5).

**Table 5:** CPM prediction capability of the development cohort, the internal and external validation.

	**0-10CMBs**	**>10 CMBs**
**Development cohort**
0-5 score	519(62.08%)	72(8.61%)
6-12 score	53(6.34%)	192(22.97%)
**Internal validation**
0-5 score	274(69.19%)	36(9.09%)
6-12 score	23(5.81%)	63(15.91%)
**External validation**
0-5 score	255(60.57%)	23(5.46%)
6-12 score	47(11.16%)	96(22.81%)

## Discussion

Currently, thrombolysis is one of the most effective treatments for AIS. sICH is the most terrible and unpredictable complication of thrombolysis. To date, there is no accurate and practicable method to predict the probability of hemorrhagic transformation. The current study, through univariate analysis and multivariate logistic regression analysis, found that history of hypertension and CSVD manifestations of CT (leukoaraiosis, lacunar infarction, brain atrophy) were independent risk factors of >10 CMBs in the brain parenchyma. A CPM for >10 CMBs was established according to the independent risk factors. The clinical efficacy of the CPM was exhibited through good discrimination, accuracy, and clinical practicability; therefore, patients can benefit from the application of the CPM. The simplified score of the CPM ranged from 0 to 12, with a cut-off value of 5 for discrimination of >10 CMBs. Through the validation, it was verified that the CPM had good clinical predictive ability and stability. Accordingly, the CPM can accurately and effectively predict the probability of >10 CMBs, thus providing an easy and practical screening tool for physicians to make clinical decisions.

CMBs are one type of imaging characteristic of CSVD (20). Neuroimaging manifestations on MRI of CSVD include (1) recent subcortical small infarct: a small (<20 mm) subcortical infarction with T1-weighted hypointensity and T2-weighted and FLAIR image hyperintensity and identified by hyperintensity on diffusion weighted imaging (DWI); (2) lacunar infarction of presumed vascular origin: a cerebrospinal fluid-filled cavity (3–15 mm) surrounded by a hyperintense rim on FLAIR images and with a signal similar to cerebrospinal fluid on all sequences; (3) white matter hyperintensities (WMH) (21) of presumed vascular origin: white matter lesions commonly distributed in the deep brain parenchyma or periventricle with hyperintensities on T2-weighted and FLAIR imaging and hypointensities on T1-weighted imaging; (4) enlarged perivascular space (EPVS) (22, 23): small, round or linear (parallel to vessels) space (<3 mm) with cerebrospinal fluid-like signal on all MRI sequences without a hyperintense rim on T2-weighted or FLAIR imaging; (5) CMB (24): small (2–10 mm) hypointensity on SWI but no corresponding signal on other conventional MR imaging; and (6) brain atrophy: local or entire cortex. As a necessary examination for patients with AIS before thrombolysis, a CT scan also reveals some CSVD imaging features such as leukoaraiosis, brain atrophy, lacunar infarction, and recent small infarction. It has been suggested that standardized visual rating scales of leukoaraiosis, lacunar infarction, and brain atrophy display good agreement between CT and MRI (25); accordingly, the burden of CSVD can be speculated using CT scan results.

CMBs often present in the area of the basal ganglia and pons, where intracerebral hemorrhage of presumed hypertensive origin typically occur (26); another location of CMBs is the subcortical region, often resulting from cerebral amyloid angiopathy (27–29). The main pathological mechanisms of CMB are considered to be hypertensive microangiopathy (lipohyalinosis and fibrinoid necrosis) and cerebral amyloid angiopathy (30); this causes destruction of the vessel wall, microaneurysm formation, and blood-brain barrier damage (31). These findings are consistent with the vascular pathological changes of sICH (32, 33). Moreover, CMB (34) is considered to be an early warning signal of intracerebral hemorrhage. In addition, clinical vascular events can occur when CMBs burden reaches a certain degree (35), and it has been reported that >10 CMBs can accurately predict sICH risk (36).

In recent years, it was reported that CSVD is a dynamic, whole-brain disorder (37); these types of CSVD often coexist when the disease is advanced. First, CSVD effects on the whole brain interstitial fluid produce subtle changes in normal white matter (38), leading to white matter hyperintensity formation (39). Next, WMHs progress, leading to secondary cortical thinning, after which acute small subcortical infarcts might appear. Finally, these cause WMH, lacunar infarctions, microbleeds, secondary cortical thinning, and worsening of long tract degeneration (40), thus leading to a heavier burden of CSVD and higher possibility of coexisting CMBs (41). In the present study, 26.80% of participants manifested the coexistence of leukoaraiosis, lacunar infarction, cerebral atrophy, and CMBs. The burden of CSVD revealed on the CT scan may indicate the number of CMBs.

All types of CSVD presenting in neuroimages correlate with each other in disease pathogenesis (42). Furthermore, CMBs and leukoaraiosis may have the same risk factors, such as hypertension (43). Poels et al. (44) confirmed that the presence of lacunar infarction and leukoaraiosis were associated with microbleeds in the deep brain parenchyma. Additionally, some investigations (45, 46) demonstrated that leukoaraiosis is a strong predictor of cerebral microbleeds. Brain atrophy frequently occurs together with WMH in elderly patients (47). Some studies demonstrated that increased hyperintensities in the deep brain parenchyma or periventricle accelerate brain atrophy (48). In addition, Yamada et al. (49) found that high-grade leukoaraiosis was a significant independent predictor for CMBs and that leukoaraiosis grade was strongly associated with the number of CMBs. However, there was no report on the relationship between CMB burden and CSVD total load, especially the CSVD manifestation of CT. In the current study, CT scan markers of CSVD were graded. We found a significant correlation of >10 CMBs with leukoaraiosis grade, brain atrophy, and lacunar infarction; this relationship was more obvious when the grade leukoaraiosis was ≥2, brain atrophy ≥2, and lacunar infarction ≥2.

Previous studies have shown that CMB was associated with age, hypertension (45), diabetes (50), coronary heart disease, history of stroke (51), smoking, and alcohol consumption (52). Age and hypertension were the strongest risk factors for CMBs. The detection rate of CMBs increased with age and was extremely low in young patients, 6.5% in patients aged 45–50 years, and 35.7% in patients aged ≥80 years (44, 53). The incidence of CMBs tends to increase with aging, and the risk of developing CMBs increases by 3% for each additional year of age (40). However, Benedictus et al. found that after adjusting for the interference of risk factors, there was no significant correlation between CMBs and age (54). In the current study, the mean age of the >10 CMB group was higher than that of the <10 CMB group, and the difference was statistically significant in univariate analysis; however, age was not an independent risk factor in the multivariate logistic regression analysis. The relationship between the number of CMBs and age needs to be explored further. Hypertension results in continued damage to smooth muscle cells and arteriolar injury; long-term hypertension can cause abnormalities in arterioles, such as arteriolosclerosis, lipohyalinosis, fibrinoid necrosis, and blood extravasation, leading to lacunar infarction, WMH, CMBs, and cerebral hemorrhage (47). CMBs and hypertensive intracerebral hemorrhage are often located similarly at the basal ganglia, pons, and cerebellum, indicating a common pathogenesis. It is well known that in addition to intracerebral hemorrhage, hypertension is an important risk factor in the development of CMB (55).

It takes a relatively short time for neurologists to evaluate CT scans and consult the hypertension history of the patient. By interpreting CT scans and inquiring about the medical history, neurologists can quickly evaluate patients with AIS. When the CPM sum score is more than to 5, the patients will be more likely to have >10 CMBs. In the case of AIS, the CPM may be a useful tool to assess the likelihood of the presence of >10 CMBs and provide a more accurate prediction of hemorrhagic transformation to guide thrombolytic therapy (56).

### Limitations

The CPM needs to be further validated externally in more medical institutions at different levels to verify the repeatability and universality of the model. Although hypertension is a recognized risk factor for CMBs, this study did not consider the grade and duration of hypertension. Furthermore, previous antiplatelet therapy and antiplatelet duration may affect CMB burden, but this was not discussed in this study. Maria et al. (2) found that the distribution of CMBs was significantly associated with sICH. Conversely, some studies found no significant correlation between the location of cerebral microbleeds and sICH (7). Therefore, the CMB anatomical distribution was not considered in the establishment of CPM. However, the relationship between CMB distribution and sICH remains to be further studied.

### Conclusions

We established a simple, easily implemented operating scoring scale. When the CPM sum score is more than 5, the patient will be more likely to have >10 CMBs. Neurologists can quickly screen patients at high risk of hemorrhagic transformation without the use of MRI, guiding thrombolysis treatment and reducing the occurrence of sICH after intravenous thrombolytic therapy.

## Data Availability Statement

The original contributions presented in the study are included in the article/Supplementary Material, further inquiries can be directed to the corresponding author/s.

## Ethics Statement

The studies involving human participants were reviewed and approved by Tangshan GongRen Hospital. Written informed consent for participation was not required for this study in accordance with the national legislation and the institutional requirements. Written informed consent was not obtained from the individual(s) for the publication of any potentially identifiable images or data included in this article.

## Author Contributions

YoL, HM, and YiL analyzed the data and drafted the manuscript. HG, DZ, XG, LL, CL, QL, and CM acquired the data. All authors read and approved the submitted version.

## Conflict of Interest

The authors declare that the research was conducted in the absence of any commercial or financial relationships that could be construed as a potential conflict of interest.

## Publisher's Note

All claims expressed in this article are solely those of the authors and do not necessarily represent those of their affiliated organizations, or those of the publisher, the editors and the reviewers. Any product that may be evaluated in this article, or claim that may be made by its manufacturer, is not guaranteed or endorsed by the publisher.
